# E‐Cadherin Is a Structuring Component of Invadopodia in Pancreatic Cancer

**DOI:** 10.1111/jcmm.70608

**Published:** 2025-05-14

**Authors:** Aurélie Dobric, Sébastien Germain, Françoise Silvy, Rénaté Bonier, Stéphane Audebert, Luc Camoin, Nelson Dusetti, Philippe Soubeyran, Juan Iovanna, Véronique Rigot, Frédéric André

**Affiliations:** ^1^ Pancreatic Cancer Team Centre de Recherche en Cancérologie de Marseille (CRCM), Institut Paoli‐Calmettes, Aix‐Marseille Université, Inserm, CNRS Marseille France; ^2^ Marseille Proteomics Platform, CRCM Institut Paoli‐Calmettes, Aix‐Marseille Université, Inserm, CNRS Marseille France

**Keywords:** adhesion molecules, cell invasion, EMT hybrid cells, matrix degradation

## Abstract

The appearance of hybrid epithelial–mesenchymal (E/M) cells expressing E‐cadherin is favourable for the establishment of pro‐invasive function. Although the potential role of E‐cadherin in cancer invasion is now accepted, the molecular mechanisms involved in this process are not completely elucidated. To gain further insight, we focused our analysis on invadopodia formation, an early event in the invasion process. We used models of E/M hybrid cell lines, tissue sections and patient‐derived xenografts from a multi‐centre clinical trial. E‐cadherin involvement in invadopodia formation was assessed using a gelatin‐FITC degradation assay. Mechanistic studies were performed by using proteomic analysis, siRNA strategy and proximity ligation assay. We showed that E‐cadherin is a critical component of invadopodia. This unexpected localization results from a synergistic trafficking of E‐cadherin and MT1‐MMP through a Rab vesicle‐dependent pathway. Modulation of E‐cadherin expression or activation impacted invadopodia formation. Moreover, colocalization of E‐cadherin and Actin in “ring structures” as precursors of invadopodia reveals that E‐cadherin is required for invadopodia structuration. E‐cadherin, initially localised in the adherens junctions, could be recycled to nascent invadopodia where it will interact with several components enriched in invadopodia, such as Arp2/3, Cortactin or MT1‐MMP. The trans‐adhesive properties of E‐cadherin are therefore essential for structuring invadopodia. This new localisation of E‐cadherin and its unexpected role in cell invasion shine a new light on hybrid E/M transition features in tumoral invasion.

**Abbreviations**
ECM: Extracellular Cellular Matrix
EMT: Epithelial‐to‐Mesenchymal transition
E/M: Hybrid Epithelial/Mesenchymal
IPA: Ingenuity Pathway Analysis
MMPs: Matrix metalloproteinases
MT1‐MMP: membrane type 1 matrix metalloproteinase
PDAC: Pancreatic Ductal Adenocarcinoma
PLA: Proximity Ligation Assay

## 1 Introduction

Pancreatic ductal adenocarcinoma (PDAC) is a cancer with a poor prognosis [1]. This aggressivity is due to a combination of factors including a lack of early diagnostic markers, a lack of symptoms and an early metastatic spread. Moreover, the desmoplastic reaction observed in PDAC is a hallmark of disease progression and prognosis. It is correlated with inflammation and low vascularity. Additionally, the presence of large amounts of extracellular matrix (ECM) components and tumour‐infiltrating leukocytes is determinant in epithelial‐to‐mesenchymal transition (EMT) evolution and therapeutic resistance [2, 3].

EMT is a key biological process associated with the gain, either individually or collectively, of mesenchymal features and the acquisition of migration and invasion properties by cancer cells, conferring metastasis properties [4]. During this process, epithelial cells lose their apical‐basal polarity, remodel their cytoskeleton and exhibit reduced cell–cell adhesion properties [5]. However, although reduced, these intercellular interactions remain essential for collective cell migration. Several studies highlight the role of the cell–cell adhesion molecule as classical cadherin in the collective migration of tumour cells [6, 7].

Studies have shown that EMT is a process with distinct intermediates, reflecting a progressive acquisition and loss of mesenchymal and epithelial molecular traits. Such traits coexist in intermediate states, as documented by a mixture of epithelial and mesenchymal features at molecular and morphological levels [8, 9]. In cancer, a hybrid E/M signature is an indicator of poor prognosis, as cancer cells may metastasize with a partial loss of epithelial and a partial gain of mesenchymal traits [8, 10, 11, 12]. PDAC encompasses a range of E/M hybrid cells, reflecting epithelial–mesenchymal plasticity [8, 10, 11, 12].

Classical cadherins establish adhesion between neighbouring cells through their extracellular domain and ensure the cohesion required for tissue integrity [13]. Their intracellular domain is associated with catenins, which allow connection to the actin cytoskeleton and cell signalling pathways. Complete downregulation of epithelial E‐cadherin associated with an up‐regulation of N‐cadherin and/or P‐cadherin during the EMT process was historically considered a crucial step in carcinoma progression to promote invasion and metastasis [14]. However, a correlation between low E‐cadherin expression, cell invasion and metastasis is not absolute. Indeed, studies have shown that tumour cells with epithelial traits and still expressing E‐cadherin can undergo metastasis and form secondary tumours [15, 16, 17]. According to this, E‐cadherin was reported as a promoter of metastasis in models of invasive ductal breast carcinomas [18, 19]. Moreover, it has been shown that some PDAC cells with high E‐cadherin expression at the cell boundaries exhibit highly invasive and malignant behaviour [16]. However, the molecular mechanisms involved in this process remain to be elucidated.

During metastasis progression, invading cells acquire the capability to degrade the extracellular matrix and ultimately invade the vasculature. These processes are driven by membrane protrusions called invadopodia characterised by an actin core, sites of cell‐ECM adhesion and by the focal degradation of ECM via matrix metalloproteases (MMPs) [20, 21, 22]. Invadopodia have been extensively studied in cell culture and have been detected in vivo [23, 24]. The assembly and function of invadopodia are regulated by a complex protein network that governs their formation, stability, and activity [20]. Invadopodia are not made up of proteins specific to this membrane protrusion. However, molecularly, invadopodia are defined as membrane structures enriched in invadopodia‐related proteins, including the scaffold protein Tks5, the actin regulators Cortactin, Wiskott–Aldrich syndrome protein family members, cofilin and membrane type 1 matrix metalloproteinase (MT1‐MMP) [20]. According to this, cortactin/Tks5 and MT1‐MMP colocalisation is a clear indicator of invadopodia. However, other as yet unidentified molecules could play a role in structuring invadopodia. MT1‐MMP is a key element in invadopodia activity, either by activating MMP‐2 or by degrading the extracellular matrix itself, leading to a restricted zone of degradation. Conversely, its role outside the invadopodia leads to massive degradation. Although considerable progress has been made in understanding the regulation of invadopodia formation and activity, a putative role of cadherins in invadopodia organisation remains unknown. Therefore, we explored how E‐cadherin modulates the ability of pancreatic cancer cells to degrade ECM.

## 2 Material and Methods

### 2.1 Antibodies and Reagents

Mouse anti‐E‐cadherin (M168 and HECD‐1), rabbit anti‐E‐cadherin (EP700Y) and anti‐Arp3 (EPR110429) were from Abcam. Mouse anti‐E‐cadherin (24E10), rabbit anti‐P‐cadherin (21300S) and rabbit anti‐Rab7 (D95F2) were from Cell Signalling. Mouse anti‐Cortactin p80/p85 (4F11) and anti‐MT1‐MMP (LEM 2/15.8) were from Millipore. Goat anti‐E‐cadherin was from St John's Laboratory. Rabbit anti‐Rab11 and rhodamine‐conjugated phalloidin were from Life Technologies. Rabbit anti‐Tks5 was from Novus. Alexafluor 488, 594, and 647 secondary antibodies were from Thermo Fisher. Both AS9 (BAS00132635) and AS11 (BAS00602705) compounds were from Asinex.

#### 2.1.1 Cell Culture

The human pancreatic adenocarcinoma BxPC‐3 cells (RRID:CVCL_0186) and SUM‐149, also named SUM149PT (RRID:CVCL_3422) cells derived from inflammatory breast cancer were purchased through the ATTC and cultured as previously published [6, 25, 26]. These cell lines were authenticated using short tandem repeat (STR) profiling within the last 3 years (STRC8012 and STRC8013). E‐cadherin was stably knocked down in BxPC3 cells using shRNA lentiviral transduction particles as previously described [6]. Primary cell cultures PDAC001T and PDAC021T derived from patient‐derived xenograft (PDX) were generated by Dusetti's team at the CRCM and routinely cultivated in SFDM as previously described [27]. E‐cadherin deficient PDAC021T were transfected with human E‐Cadherin mGFP‐tagged ORF Clone Lentiviral Particle (Origene) at 25 multiplicity of infection. Infected cells were selected using 2.5 μg/mL puromycin. E‐cadherin expression was checked by western blot analysis. All experiments were performed with mycoplasma‐free cells.

#### 2.1.2 Reverse siRNA Transfection

ON‐target plus smartpool human control, human MT1‐MMP, human Arp3, human Rab7A and human Rab11A were from Dharmacon. Cells were seeded in 6‐well plates directly with the siRNA/transfection mix: 3 μL of LipoRNAiMax (Life Technologies), 500 μL of OptiMEM (Life Technologies), 25 or 50 nM of indicated siRNA and 2.5 mL of RPMI/10% FCS medium. When required, transfected cells were detached and seeded on FITC‐gelatin coated coverslips, 24 or 48 h after treatment.

#### 2.1.3 Subcutaneous Xenografts of Pancreatic Cancer Cells

BxPC‐3 cells were harvested by mild trypsinisation, washed twice in PBS, then suspended in Matrigel at 2 × 10^6^ cells per 100 μL. To induce tumours, the cell suspension was injected subcutaneously (s.c.) into the flank of 6–8‐week‐old female NMRI‐Foxn1nu/Foxn1nu mice (Charles River Laboratories, L'Arbresle, France). For injections of tumour cells, the animals were chosen at random. Mice were sacrificed 3 weeks after inoculation. Tumours were removed and tissue specimens were fixed in 4% formalin, then embedded in paraffin.

#### 2.1.4 Immunohistofluorescence

Human tissue specimens or mouse tumours were cut into 3 μm sections. After dewaxing and antigen retrieval at pH 9, sections were incubated for double or triple staining with primary antibodies for 2 h at room temperature. After washing, the sections were incubated with Alexa Fluor‐conjugated antibodies, washed and mounted in aqueous mounting medium. Images were captured with an LSM 880 Zeiss confocal microscope equipped with ZEN Software (objective 40×). Colocalization quantifications were performed using Jacop plugging (FiJi software). Overlap coefficients were obtained by dividing the number of points in the overlap region (different channels) by the total number of points in one of the distributions (each channel).

#### 2.1.5 Invadopodia Assay

Coverslips were coated with FITC‐conjugated gelatin (Life Technologies), fixed with 0.5% glutaraldehyde and incubated for 3 min at RT with 5 mg/mL sodium borohydride (Sigma). After washes, 10^4^ isolated cells were seeded on top of the coverslip. Cells were incubated for 16 h at 37°C in their appropriate medium (DMEM/10%FCS, RPMI/10%FCS or SFDM). Cells were fixed in 4% formaldehyde, then permeabilized and blocked with phosphate buffered saline/bovine serum albumin (PBS/BSA) 4% saponin 0.1% for 1 h. Cells were successively incubated with indicated primary antibodies in PBS/BSA 1% saponin 0.1% for 2 h at RT and with Alexa Fluor‐conjugated secondary antibodies in PBS/BSA 1% saponin 0.1% for 1 h raised against mouse or rabbit immunoglobulins (Invitrogen). After washes, samples were mounted in ProLong Gold antifade reagent (Thermo Fisher Scientific). The areas of degraded matrix were observed using Sp5 Leica or a LSM880 Zeiss confocal microscope (20× objective). Z‐Stack acquisitions (range: 0.5 μm) were performed using a 63× objective magnification and analysed through orthogonal projections using ImageJ software (rsb.info.nih.gov/ij/). 15 microscopic fields (at least 5 cells/field) per coverslip and two coverslips per condition were acquired with all fluorescent channels. Actin and E‐cadherin ring structures were obtained using the ZEIS LSM880 AiryScan 2.5D module.

#### 2.1.6 Kinetic of Invadopodia Formation

Lab‐Teck chamber coverglasses were coated as described for invadopodia assays. 2 h after seeding, cells were incubated for 18 h with AS11 (0.01 mM) or DMSO in a temperature‐and CO_2_‐controlled chamber mounted on an Olympus IX83 inverted microscope. Cells were then washed and incubated in DMEM/10% FBS for an additional 24 h period. Invadopodia formation was analysed by videomicroscopy by capturing images every hour using an orca‐flash4 camera with a 40X objective.

#### 2.1.7 Immunoprecipitation

BxPC‐3 cells were plated in 10 cm^2^ culture dishes. Subconfluent cells (70% of confluence) were lyzed on ice with lysis buffer (50 mM HEPES pH 7.5; 150 mM NaCl; 1 mM EDTA; 1 mM EGTA; Glycerol 10%; Triton X‐100 1%; 25 mM NaF; 10 μM ZnCl_2_ + protease inhibitor cocktail). Protein G Sepharose beads (Roche) were pre‐incubated with 1 μg of the indicated primary antibody for 2 h at 4°C. After washes, equal amounts of cell lysate were incubated with the pre‐incubated beads for 2 h at 4°C. After three washes in PBS, immunoprecipitated proteins were solubilised in Laemmli buffer, heated at 100°C for 5 min, and analysed by western blotting.

#### 2.1.8 Western Blotting

Cells were lyzed with 150 mM RIPA Buffer (25 mM Tris–HCl pH 8.0; 150 mM NaCl; 1% Triton‐X100) containing protease inhibitor cocktail. Equal amounts of cell lysate (25 μg) were resolved by SDS PAGE (8% or 10% polyacrylamide) and blotted onto a polyvinylidene difluoride (PVDF) membrane. Proteins were detected using indicated antibodies. Antigen–antibody complexes were revealed using the ECL detection system (Millipore) and detected using a Pxi imaging device (SynGene).

#### 2.1.9 Invadopodia Fractioning

The isolation of an enriched fraction of invadopodia was performed using a previously published protocol [28]. Cells were seeded at 2.5 × 10^5^ cells on 4 culture dishes (10 cm diameter) coated with non‐fluorescent gelatin. After 18 h, plates were washed in PBS containing 0.5 mM MgCl2, 1 mM CaCl2, then in five‐times diluted PBS containing 0.5 mM MgCl2, 1 mM CaCl2, and incubated for 15 min in the presence of 3 mL of the diluted PBS containing a protease inhibitor mixture to induce cell swelling. Cell bodies were then sheared away using an L‐shaped Pasteur pipette with a sealed end to leave invadopodia embedded in the gelatin. The embedded invadopodia were then washed in PBS containing 0.5 mM MgCl2, 1 mM CaCl2 until no cell bodies were visible on the dishes. Then the embedded invadopodia were scraped away with the gelatin into lysis buffer (150 mM NaCl, 1% NP40, 0.5% sodium deoxycholate, 0.1% sodium dodecyl sulphate, 50 mM Tris base buffer pH 8, proteases inhibitor) and clarified by centrifugation (15 min, 13,000 rpm at 4°C). The cell body fraction was further separated into cell body membranes and cytosol fractions by centrifugation at 9000 ***g*** for 20 min at 4°C. The supernatant (cytosolic fraction) was discarded whereas the cell body membrane pellet obtained after centrifugation was solubilised in lysis buffer and clarified by centrifugation (15 min, 13,000 rpm at 4°C). Both invadopodia and cell membrane fractions were precipitated in 3 volumes of cold acetone overnight at −20°C, centrifuged and denatured. All the invadopodia membrane fraction corresponding to 4 dishes was loaded with 1/2 of the cell body membrane fraction.

#### 2.1.10 Proximity Ligation Assay

Proximity ligation assay (PLA) was performed according to the manufacturer's recommendations protocol (Duolink; Sigma). Briefly, cells were prepared as for indirect immunofluorescence. Cells were incubated with indicated primary antibodies for 2 h at RT. After washing, samples were incubated with the respective PLA probes (Duolink in situ probes anti‐Rb PLUS and Duolink in situ probe anti‐Mouse MINUS) for 1 h at 37°C, washed and then ligated for 30 min at 37°C. Amplification with polymerase was then performed for 100 min at 37°C in the dark. After washes, nuclei were stained with DAPI, and samples are mounted in ProLong Gold antifade reagent. Images were captured as described in the indirect immunofluorescence staining section.

#### 2.1.11 Mass Spectrometry Analysis

Proteomic analysis from E‐cadherin depleted cells (BxPC‐3 shEcad) was compared to control cells (BxPC‐3 shCTRL) by label‐free quantitative mass spectrometry analysis. Briefly, we used 15 μg of each cell lysate for proceeding and trypsin digestion [29, 30]. Details of sample preparation and data processing protocols are available in Data S1.

#### 2.1.12 Pathway Enrichment Analysis

Proteins identified by mass spectrometry were analysed with Ingenuity Pathway Analysis (IPA) software to study pathway enrichment. The statistical significance of the enrichment was calculated using the FDR method (*p*‐value < 0.05). Protein Z‐score was calculated for each protein of the selected pathway. The Z‐score indicates the overall activation state.

#### 2.1.13 Statistics

Data are presented as the mean ± SEM for three independent experiments performed in triplicate. Comparison between two conditions was made using the two‐sided Mann–Whitney test: *p* < 0.05 was considered statistically significant in all analyses and is indicated by “***” when *p* < 0.001, “**” when *p* < 0.01 and “*” when *p* < 0.05.

## 3 Results

### 3.1 E‐Cadherin Localises Within Invadopodia

We previously described that both E‐cadherin and P‐cadherin are jointly expressed at the cell surface of tumoural cells in a large proportion of PDAC, pointing to the importance of hybrid E/M cells expressing E‐cadherin in this pathology [6]. Previous studies have shown that E‐cadherin at the cell boundaries exhibits highly invasive and malignant behaviour [16]. To strengthen this function in pancreatic cancer, we used in vitro approaches to determine how E‐cadherin regulates cell invasion by analysing the formation of invadopodia, an early step of the invasion process.

The hybrid E/M BxPC‐3 cell model was first used since it expresses high levels of E‐cadherin [6]. X–Z confocal projections showed localization of actin spots with several markers enriched in invadopodia (Cortactin, Tks5 and MT1‐MMP) within a degradation area of the FITC‐labelled gelatin (Figure S1A–C). Moreover, MMP inhibitors (Figure S1D,E) and MT1‐MMP depletion by siRNA strategy (Figure S1F,G) almost entirely reduced both the capacity of these cells to degrade gelatin and the number of cells forming invadopodia. MT1‐MMP depletion using siRNA strategy was controlled by western blot (Figure S1H). This indicates that the hybrid E/M BxPC‐3 cell model exhibits active invadopodia.

**FIGURE 1:**
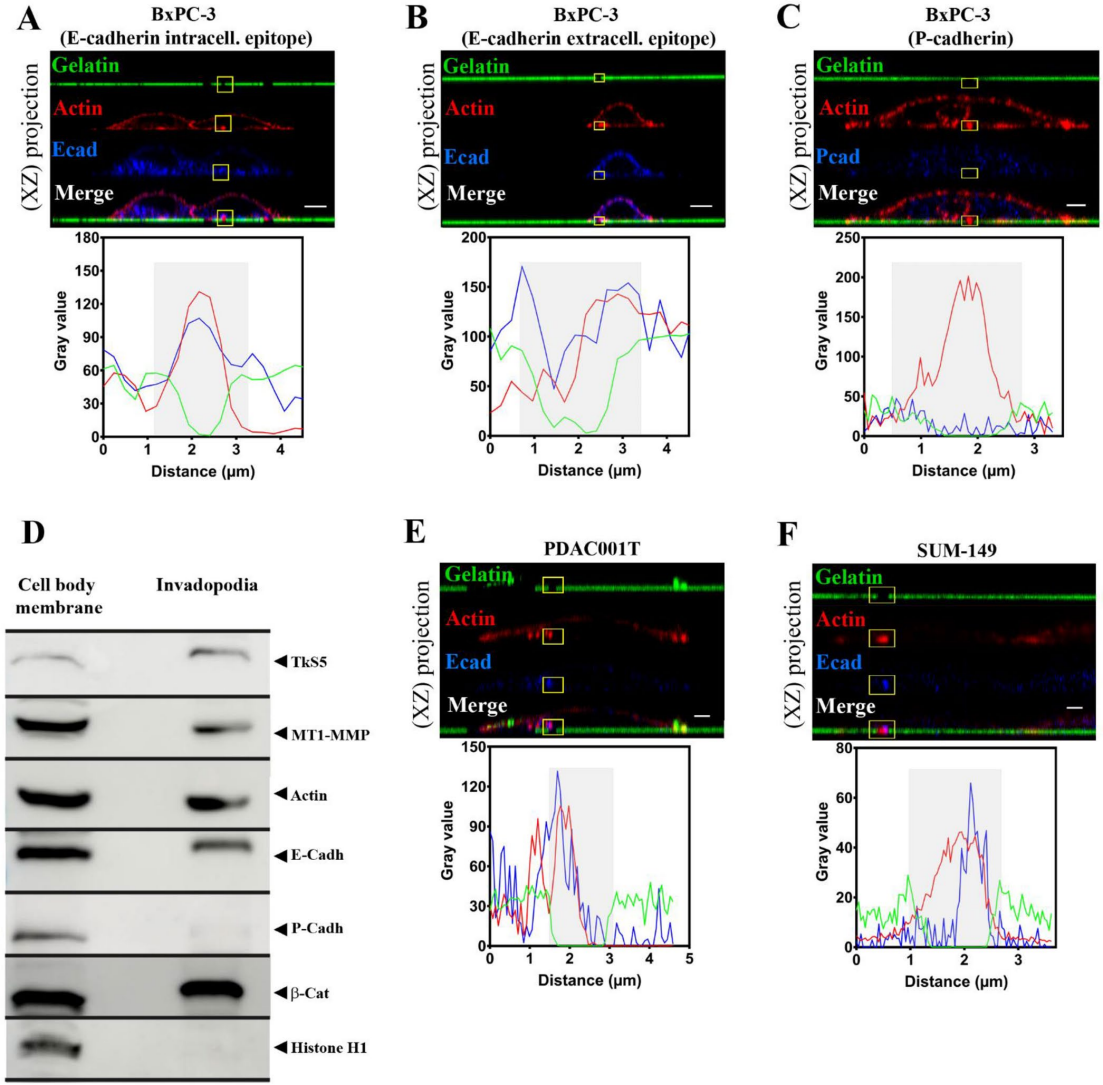
E‐cadherin localises within invadopodia. Pancreatic cancer BxPC‐3 cell line (A–C), pancreatic cancer primary culture PDAC001T (E) and breast cancer cells SUM‐149 cell line (F) were seeded on FITC‐labelled gelatin. Cells were stained for actin with phalloidin‐rhodamin (red) and E‐cadherin using an antibody raised against the cytoplasmic domain (blue) (A) or extracellular domain (B) or P‐cadherin (C). An actin spot localization with a degradation zone of the FITC‐labelled gelatin represents an active invadopodia. Top panel: Images represent Z‐stack confocal acquisitions. Scale bar = 10 μm (A, B) or 2 μm (C, E, F). Bottom panel: Fluorescence intensity quantification of the region of interest indicated by the yellow square on the top panel. The gelatin degradation area is identified in grey. For each condition, at least 50 cells were observed. (D) The BxPC‐3 cell body membrane and invadopodia membrane were enriched as described in Methods, subjected to SDS‐PAGE and transferred onto nitro‐cellulose membrane. TkS5, E‐cadherin, MT1‐MMP, P‐cadherin, Actin and Histone H1 were sequentially detected by western blot in the same membrane. Images in 2D view for (A) and (C) are available in Figure S3A. (A): A representative image of 7 experiments with 5 acquisitions for each (*n* = 7), (B, C): A representative image of 3 experiments with 5 acquisitions for each (*n* = 3), (D): One experiment representative of 3, (E, F): A representative image of 2 experiments with 5 acquisitions (5 cells per acquisition) for each (*n* = 2).

Immunostaining analysis, using antibodies raised either against the intracellular (Figure 1A) or the extracellular domain of E‐cadherin (Figure 1B) revealed that a pool of E‐cadherin is located at the invadopodial membrane. Out of 283 invadopodia counted, 98 contained E‐cadherin, that is 34.6%. P‐cadherin, also expressed at the BxPC‐3 cell membrane [6], was never detected at the invadopodial membrane (Figure 1C), indicating a specificity of E‐cadherin localization in invadopodia. Biochemical analysis on fraction enriched in invadopodia reassuringly confirmed the presence in the invasive structure of E‐cadherin (Figure 1D).

As observed in immunostaining analysis, P‐cadherin is not detected in the invadopodia fraction by western blot and could be considered a negative control of cell membrane contamination. Histone H1 is not detected in the invadopodia fraction as a negative control for cell body contamination (Figure 1D).

E‐cadherin was also detected in invadopodia of primary pancreatic cancer cells PDAC001T (Figure 1E) and SUM‐149 cell line derived from inflammatory breast cancer (Figure 1F). Therefore, the localisation of E‐cadherin in invadopodia could be extended to other cell and cancer types.

### 3.2 E‐Cadherin Localises With Cortactin and Tks5 at Invadopodia‐Like Structures in PDAC

**FIGURE 2:**
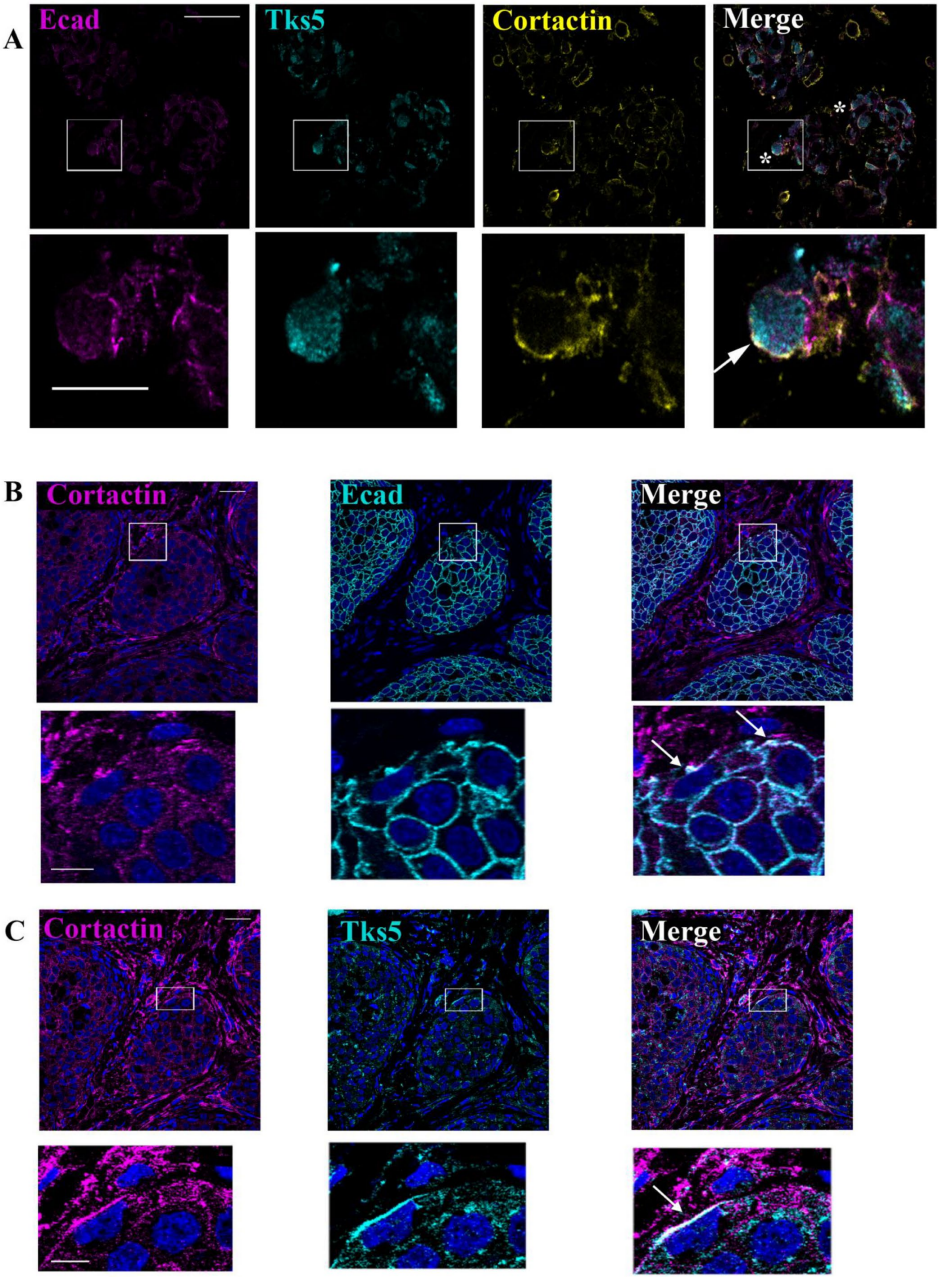
E‐cadherin localises in invadopodia‐like structures in vivo. (A) Triple E‐cadherin, Tks5, and Cortactin immunostaining in sections from patient tumours. White squares represent magnified views. White arrow indicates invadopodia containing E‐Cadherin. Scale bars represent 10 μm (top panel) or 2 μm (magnification panel). The triple Cortactin/Tks5/E‐cadherin colocalization was observed in 3 out of 6 patient tissues (*n* = 6). (B) E‐cadherin and Cortactin or (C) E‐cadherin and Tks5 double immunostaining in serial sections from subcutaneous tumours of BxPC3 cells implanted in mice. Nuclei were stained using DAPI. White squares represent magnified views. White arrows indicate spots of Cortactin, Tks5 and E‐cadherin colocalization. Scale bars represent 40 μm (top panels) or 10 μm (magnification panel).

To confirm these observations in vivo, we immunostained E‐cadherin on patient tissues, as well as Cortactin and Tks5. A Cortactin/Tks5/E‐cadherin triple staining on tissue sections revealed that a pool of E‐cadherin localises with Cortactin and Tks5 at the plasma membrane of cells localised in contact with the extracellular matrix (Figure 2A). For each structure, we quantified the overlap coefficients: for E‐cadherin/Cortactin (75.8%), E‐cadherin/Tks5 (84.6%) and Tks5/Cortactin (70.3%). This triple Cortactin/Tks5/E‐cadherin colocalisation was observed in 3 out of 6 patient tissues, indicating that invadopodia‐like structures could be detected in patient tissues.

Moreover, Cortactin or Tks5 were immunostained with E‐cadherin in human pancreatic cancer BxPC‐3 cells that had been ectopically implanted in mice. On serial tissue sections, colocalization of Cortactin/E‐cadherin and Cortactin/Tks5 was observed at tumour cell plasma membranes in close contact with the microenvironment (Figure 2B,C). Overlap coefficients are consistent with these observations: E‐cadherin/cortactin (78.2%) and Tks5/cortactin (60.2%).

Even if these structures are not common, these data confirm, in vivo, the existence of interactions between E‐cadherin and invadopodia components. This suggests the localisation of E‐cadherin inside invadopodia‐like structures.

### 3.3 E‐Cadherin Interacts With MT1‐MMP in Invadopodia and Is Recycled Through Rab7 and Rab11 Pathways

**FIGURE 3:**
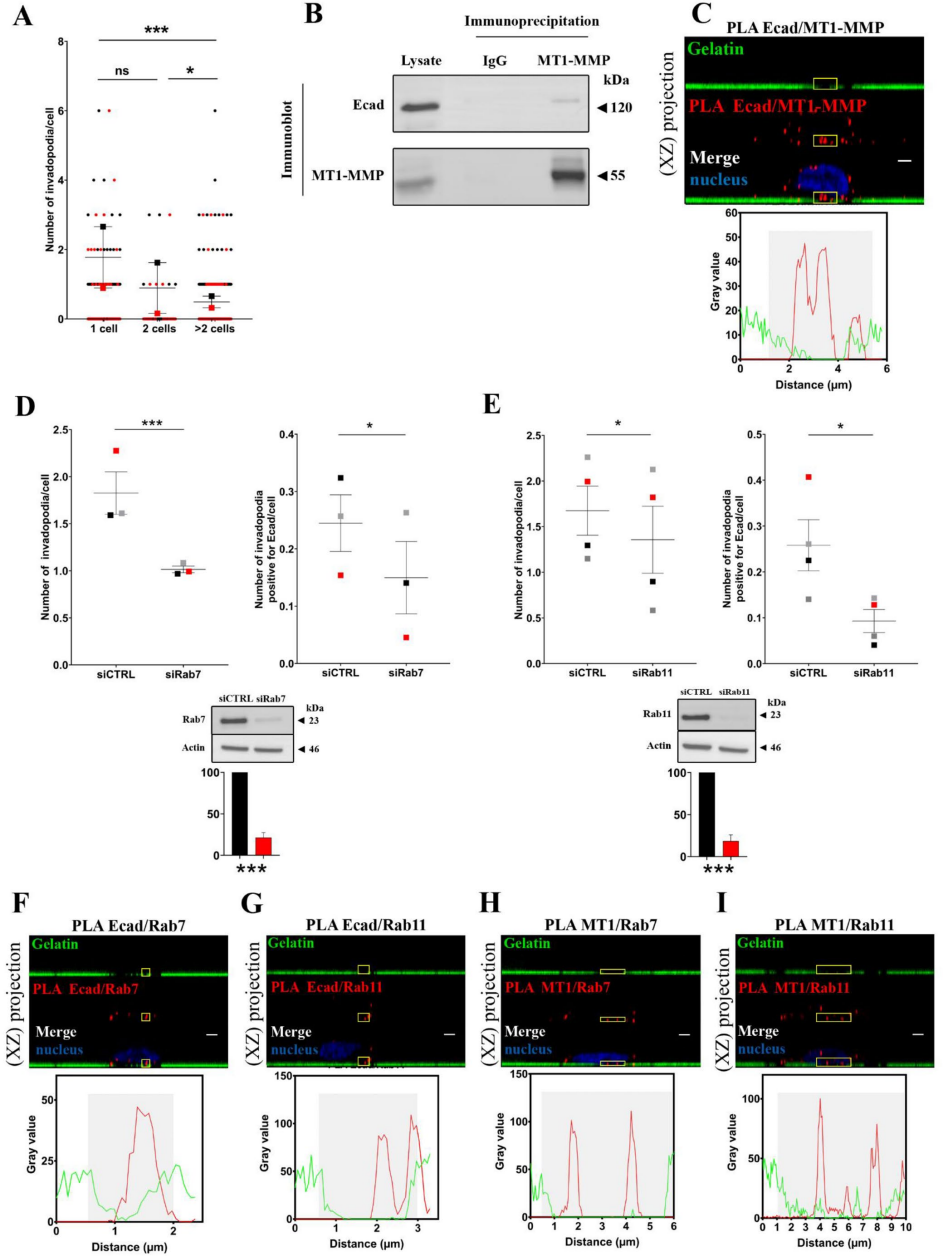
E‐cadherin interacts with MT1‐MMP in invadopodia and is recycled through Rab7 and Rab11 pathways. (A) Cell–cell interactions inhibited invadopodia formation. The number of invadopodia per cell was measured as described in Methods section. The graph represents the distribution of invadopodia in isolated cells (1 cell), cell doublet (2 cells) or groups superior of 2 cells (> 2 cells). Raw data are shown with coloured dots. Mean from 2 independent experiments are indicated with coloured squares. Errors bars represent mean ± SEM. *n* = 2. (B) Equal amounts of BxPC‐3 cell lysate were immunoprecipitated using either anti‐MT1‐MMP or non‐specific (IgG) antibodies. After SDS‐PAGE and transfer onto PVDF membrane, protein complexes were detected using anti‐E‐cadherin or anti‐MT1‐MMP antibodies. Control was performed using BxPC‐3 lysates. A representative experiment of 3 (*n* = 3) (C) E‐cadherin and MT1‐MMP colocalize inside invadopodia. After E‐cadherin and MT1‐MMP immunostaining, E‐cadherin–MT1‐MMP complexes were detected using PLA. Z‐stack confocal acquisitions were performed. Top panel: The amplification spots (in red) localise in a gelatin‐degradation area. Scale bar = 2 μm. Bottom panel: Fluorescence intensity quantification of the region of interest indicated by the yellow square on the top panel. The gelatin degradation area is identified in grey. A representative image of 2 experiments in triplicates with 3 acquisitions for each (*n* = 2). **(**D, E) BxPC‐3 cells were treated for 48 h with siRNA control (siCTRL) or siRNA against (D) Rab7 (siRab7) or (E) Rab11 (siRab11) before invadopodia assay. (D, E) Left panel: Quantification of active invadopodia at the ventral surface of each cell. Right panel: Quantification of active invadopodia exhibiting E‐cadherin per cell. Means from 3 (D) or 4 (E) independent experiments indicated with coloured squares. Errors bars represent mean ± SEM. Bottom panels: Equal amounts of cell lysate (25 μg) were subjected to SDS‐PAGE, then transferred onto PVDF membrane. Graphs represent the mean ± SEM of Rab7 or Rab11 protein expression from 3 independent cell transfection. (F) E‐cadherin and Rab7; (G) E‐cadherin and Rab11; (H) MT1‐MMP and Rab7; (I) MT1‐MMP and Rab11. Z‐stack confocal acquisitions were performed on fixed cells. Left panels: The amplification spots (red) localise with a degradation spot of the fluorescent gelatin (green). Scale bars represent 2 μm. Right panels: Fluorescence intensity quantification of the regions of interest indicated by the yellow square on the left panel. Images in 2D view for (C) and (F–I) are available in Figure S3B and negative control (PLA probe PLUS/MINUS) is available in Figure S3D. (F–I): A representative image of 2 experiments in triplicates with 3 acquisitions for each (*n* = 2). ***:*P* < 0.001; *:*P* < 0.05.

To understand if this surprising localization of E‐cadherin depends on a random distribution, we first investigated the impact of cell–cell interaction on invadopodia formation. We observed that the number of invadopodia decreases in cells exhibiting intercellular contacts (Figure 3A), indicating a possible competition between the formation of cell–cell interactions and invadopodia. This suggests that the endocytic and exocytic fluxes of cell–cell contact components are crucial for invadopodia activity.

Vesicular transport has been shown to be crucial for invadopodia formation by facilitating the trafficking of MT1‐MMP to the plasma membrane [31]. On the other hand, E‐cadherin undergoes cycles of endocytosis, sorting and recycling to the plasma membrane through Rab7 and Rab11 vesicles [32, 33]. We therefore assessed if E‐cadherin interacts with MT1‐MMP and if its targeting to invadopodia depends on the same recycling process. Biochemical studies showed that a pool of E‐cadherin co‐precipitates with MT1‐MMP, thus suggesting that these molecules can associate together (Figure 3B). Furthermore, PLA documented E‐cadherin interaction with MT1‐MMP in different cellular localisations: cell membrane, cytoplasmic vesicles and within invadopodia structures (Figure 3C). We found that depletion of Rab7, which controls transport to late endocytic compartments (Figure 3D) or Rab11, which regulates dynamics of recycling endosomes (Figure 3E), decreases the number of invadopodia containing E‐cadherin. Moreover, E‐cadherin‐Rab7 (Figure 3F) and E‐cadherin‐Rab11 complexes (Figure 3G) were observed in several cytoplasmic vesicles, some of which were localised in the immediate vicinity of the degradation areas. MT1‐MMP was also detected in these compartments (Figure 3H,I). Altogether, these data indicate that E‐cadherin is trafficked from the plasma membrane to invadopodia via an active recycling process through Rab7 and Rab11 pathways. They also demonstrate that MT1‐MMP and E‐cadherin could interact with each other inside invadopodia and are trafficked through the same pathway.

### 3.4 E‐Cadherin Adhesive Activity Is Required for Invadopodia Formation

**FIGURE 4:**
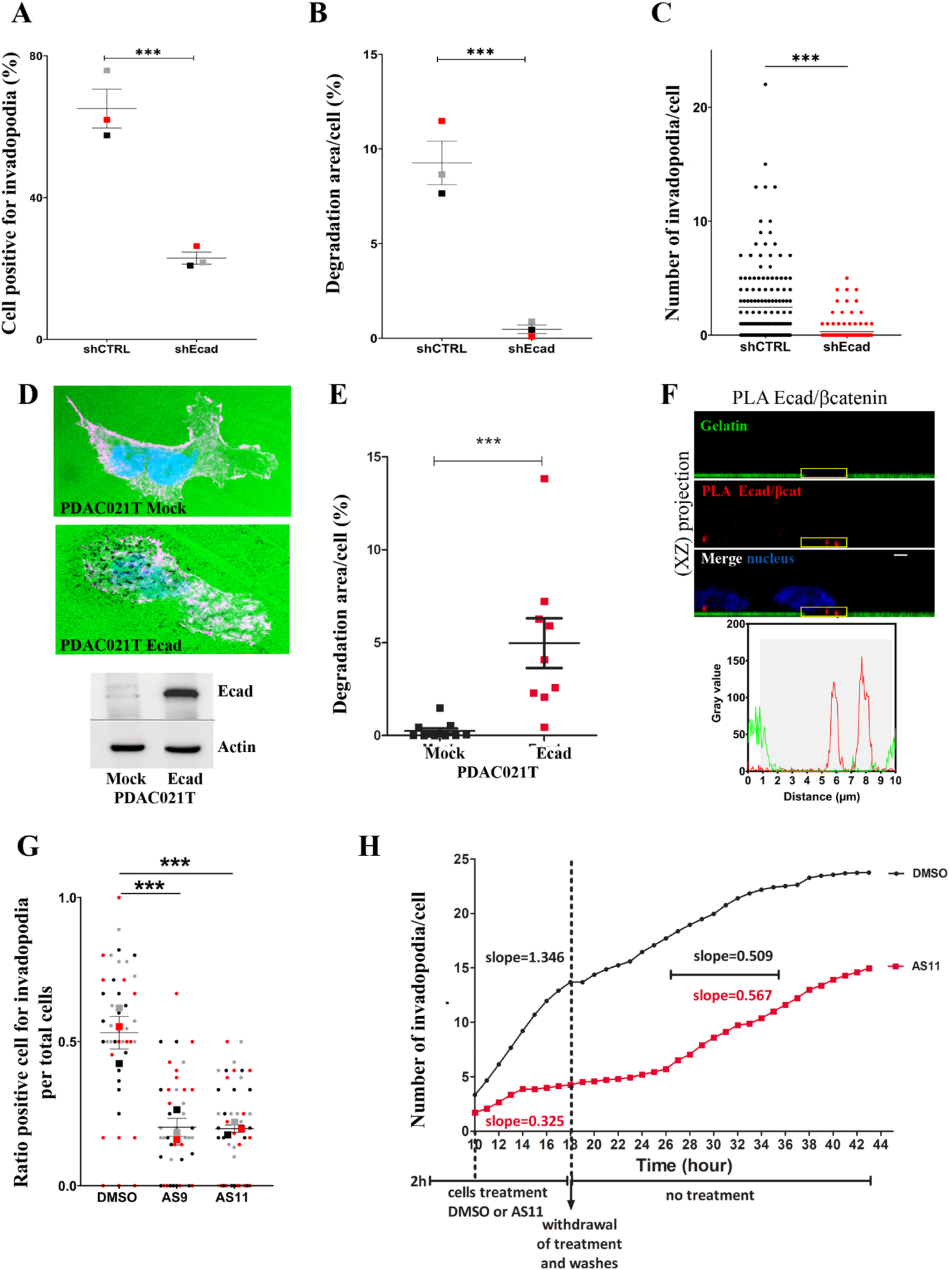
E‐cadherin adhesive activity is required for invadopodia formation. (A‐C) Invadopodia assays were performed using BxPC‐3 control (shCTRL) and E‐cadherin depleted cells (shEcad) cell lines. (A) The number of cells exhibiting active invadopodia were quantified. Means from 3 independent experiments are indicated with coloured squares. Errors bars represent Mean ± SEM. *n* = 3 (B) The normalised gelatin degradation area at the ventral surface of the cells was evaluated. Means from 3 independent experiments are indicated with coloured squares. Errors bars represent mean ± SEM. *n* = 3. (C) The distribution of the number of invadopodia per cell was determined A representative graph of 3 experiments (*n* = 3). Images in 2D view for (A) are available in Figure S3C. (D‐E) Invadopodia assays were performed using PDAC021T Mock (no E‐cadherin expression) and PDAC021T Ecad, (E‐cadherin expression) cells. The E‐cadherin expression was assessed by western blot. The normalised gelatin degradation area at the ventral surface of the cells were evaluated. Representative results from 3 independent experiments. (F) E‐cadherin and β‐catenin interact within invadopodia. E‐cadherin–β‐catenin complexes were detected using a PLA. Z‐stack confocal acquisitions were performed. Top panel: The amplification spot (red) localises in a degradation spot of FITC‐labelled gelatin (green). Bottom panel: Fluorescence intensity quantification of the region of interest indicated by the yellow square on the top panel. Scale bar represents 2 μm. A representative image of 2 experiments in triplicates with 3 acquisitions for each (*n* = 2). Images in 2D view for (F) is available in Figure S3D. (G) E‐cadherin inhibition decreases invadopodia formation. Ratio of cells exhibiting active invadopodia in treated (AS9 or AS11) and untreated (DMSO) BxPC‐3 cells were evaluated. Means from 3 independent experiments are indicated with coloured squares. Raw data are shown with coloured dots. Errors bars represent mean ± SEM. *n* = 3. (H) Invadopodia assays were performed using BxPC‐3 shCTRL. Cells were seeded for 2 h on coverslips coated with FITC‐labelled gelatin, then treated for 16 h with DMSO or AS11. Cells were then washed and incubated in DMEM/10% fetal calf serum for an additional 24 h period. Invadopodia formation was analysed by videomicroscopy by capturing images every hour, 8 h after addition of the compounds. The number of gelatin degradation zones appearing just below the cell body is estimated for each hour. The graph is representative of an experiment carried out three times (*n* = 3). ***:*P* < 0.001.

We next explored the role of E‐cadherin in invadopodia formation and function using a cellular system engineered to silence E‐cadherin expression by shRNA. Specifically, we generated stable BxPC‐3 shEcad (E‐cadherin depletion) and control BxPC‐3 shCTRL (no cadherin depletion) cells [6]. We found that E‐cadherin silencing promotes a significant decrease in the number of cells forming invadopodia (Figure 4A). This was accompanied by a reduction in degradation areas (Figure 4B) and a significant decrease in the number of invasive structures per cell (Figure 4C). Conversely, forced E‐cadherin expression in E‐cadherin deficient cells derived from patient tumour (Figure 4D) promotes a significant increase in degradation areas (Figure 4D,E).

E‐cadherin requires interactions with catenins to be functional. PLA indicated that E‐cadherin associates with β‐catenin within invadopodia (Figure 4F). Moreover, we found that two synthetic E‐cadherin inhibitors, AS9 and AS11, which block trans‐interactions of E‐cadherin molecules in junctional complexes [34] reduced the number of cells exhibiting invadopodia (Figure 4G). By using videomicroscopy, we analysed the impact of AS11 on the kinetics of invadopodia formation. AS11 decreased the rate of invadopodia appearance by 75% for the cells that still perform invadopodia, since the slope of the curves is 0.325 for AS11‐treated cells versus 1.346 for control cells (Figures 4H and S2). The inhibitory effect of AS11 is rescued by removing the compounds. Indeed, cells resume invadopodia formation after 8 h of latency with the same speed as control cells when the E‐cadherin inhibitor is removed. The slopes of the curves are 0.567 for the cells previously treated with AS11 and 0.509 for the control cells. These data confirm the presence of a pool of functional E‐cadherin at the invadopodial membrane and strongly suggest a role of E‐cadherin in invadopodia structuring.

### 3.5 An E‐Cadherin/Arp3 Complex Is Detected Into Invadopodia

**FIGURE 5:**
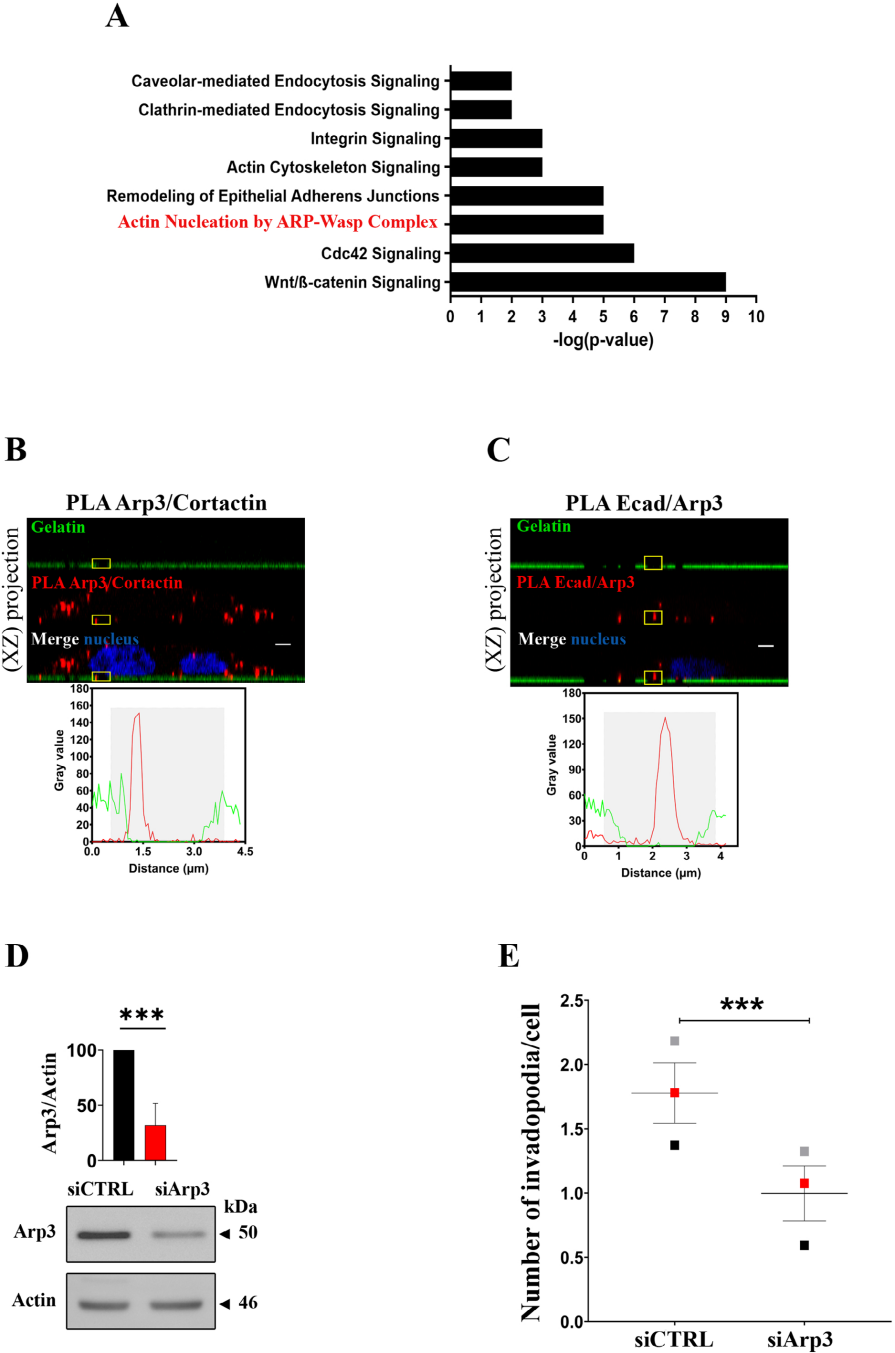
An E‐cadherin/Arp3 complex is detected into invadopodia. (A) Most deregulated signalling pathways in BxPC‐3 shEcad compared with BxPC‐3 shCTRL cells as determined by IPA analysis of proteome data. The enrichment score on the graphic is represented by −log(*p*‐value). *n* = 3 (B, C) Protein–protein interactions in invadopodia revealed by PLA. (B) Arp3–Cortactin and (C) Arp3–E‐cadherin interactions. Z‐stack confocal acquisitions were performed on fixed cells. Top panels: The amplification spots (red) localise with a degradation spot of FITC‐labelled gelatin (green). Cell nuclei are shown in blue. Bottom panels: Fluorescence intensity quantification of the region of interest indicated by the yellow square on the left panel. Scale bar represents 2 μm. (B): A representative image of 2 experiments in triplicates with 3 acquisitions for each (*n* = 2). (C): A representative image of 2 experiments in triplicates with 3 acquisitions for each (*n* = 2). Images in 2D view for (C) are available in Figure S3D and negative control for (B, C) is available in Figure S3D. (D) BxPC‐3 cells were treated for 48 h with control siRNA (siCTRL) or siRNA against the Arp3 subunit (siArp3). Arp3 protein expression in BxPC‐3 cells treated by siCTRL or siArp3. Equal amounts of cell lysate (25 μg) were subjected to SDS‐PAGE, then transferred onto PVDF membrane. Arp3 and actin were detected using specific antibodies. The graph represents the mean ± SEM of Arp3 protein expression from 3 independent cell transfection. *n* = 3. (E) After a treatment during 48 h with control siRNA (siCTRL) or siRNA against the Arp3 subunit (siArp3) cells were plated for 16 h onto FITC‐labelled gelatin. The graph represents the quantification of active invadopodia formed per cell. Data corresponds to a mean from three independent experiments indicated with coloured squares. Errors bars represent mean ± SEM. *n* = 3.

To determine if E‐cadherin expression could regulate the formation of invadopodia, we analysed by mass spectrometry the full proteome of E‐cadherin depleted BxPC‐3 cells (shEcad) versus cells expressing E‐cadherin (shCTRL). The analysis of these data using the Ingenuity Pathway Analysis (IPA) software suggests at least 8 signalling pathways deregulated upon E‐cadherin depletion, of which some could be crucial for invadopodia formation (Figure 5A). Among enriched pathways, the actin nucleation by ARP/WASP complex was particularly interesting as this complex has been described in E‐cadherin trafficking [35]. We therefore focused on this pathway and found that Arp3 associates with both Cortactin and E‐cadherin close to gelatin degradation areas, suggesting E‐cadherin/Arp3 complex implication in invadopodia structuring (Figure 5B,C).

To functionally assess the implication of E‐cadherin/Arp3 complex on invadopodia formation, we generated Arp3‐silenced cells (Figure 5D). Reassuringly, we found that Arp3 depletion promoted a significant decrease in the number of invadopodia formed per cell (Figure 5E). Altogether, these data highlight the importance of E‐cadherin in the actin nucleation process through ARP/WASP complex.

### 3.6 E‐Cadherin Is a Structuring Component of Invadopodia

The association between E‐cadherin and Arp3 promotes a signal for actin assembly during adherens junction formation [35]. If the E‐cadherin/Arp2/3 complex is involved in the structuring of invadopodia, this should be reflected in the observation of complex formation prior to matrix degradation.

**FIGURE 6:**
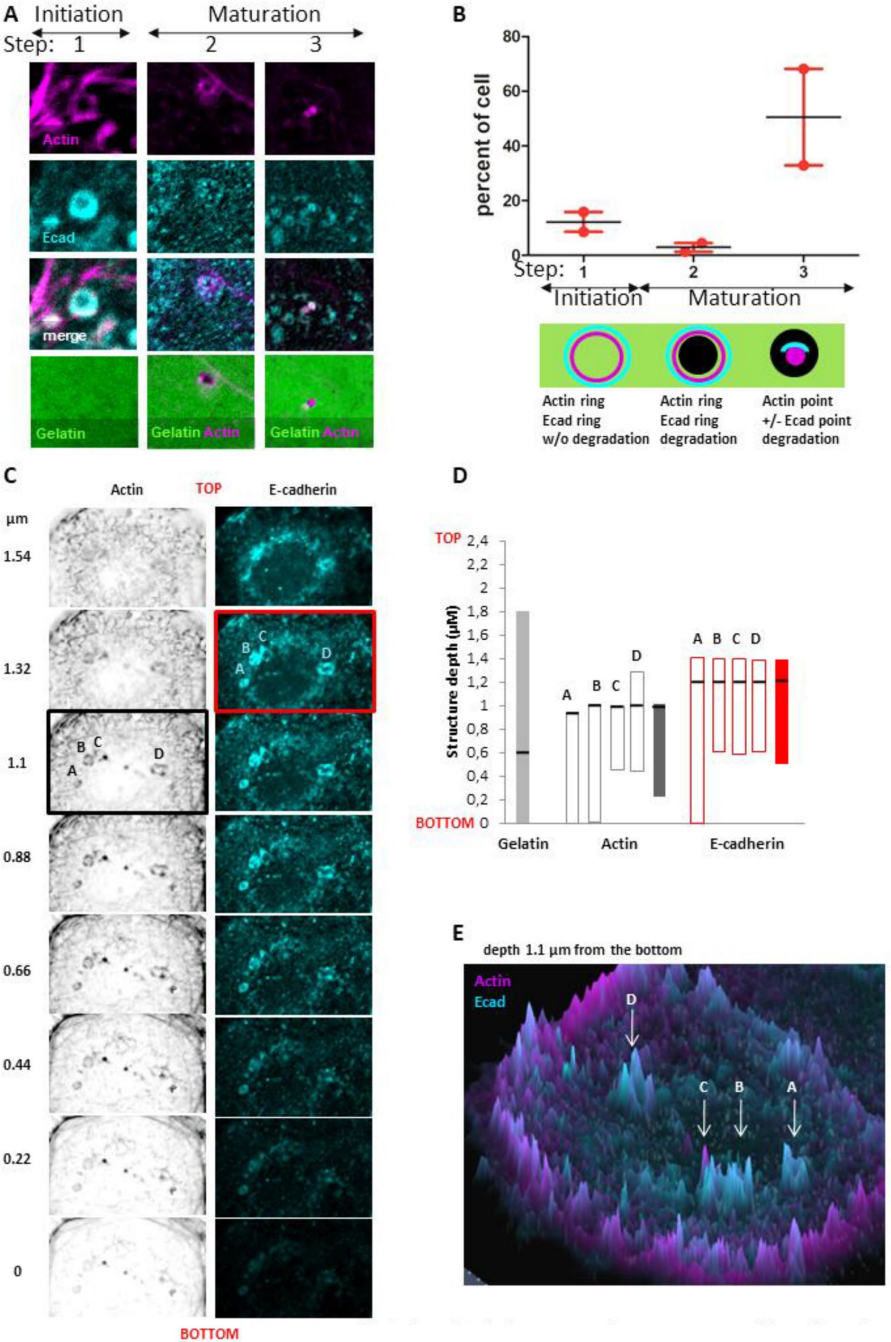
E‐cadherin is a structuring component of invadopodia. Invadopodia assays were performed as previously described. (A, B) After confocal acquisition, analysis of structures evidence 3 kinds of invadopodia: Step 1 initiation: In the absence of FITC‐labelled gelatin degradation Actin ring overlays with E‐cadherin ring, step 2 maturation: Spot of associated with both Actin and E‐cadherin ring, step 3: FITC‐labelled gelatin degradation associated with Actin spot in the presence or absence of E‐cadherin. (B), Percentage of cells showing these kinds of structure (*n* = 2). (C–F) Images of Invadopodia assays were acquired using AiryScan module of Zeiss LSM 880 confocal microscope. Acquisitions were performed inside the gelatin sheet every 0.22 μm. Bottom indicated the bottom detection of gelatin. In (C), labelling of both Actin E‐cadherin are given for each section. Intensity quantifications were performed using FIJI software and the maximal intensity for Actin and E‐cadherin is mentioned by a black or red box, respectively. In (D) Z‐labelling ranges are positioned with bars and the section with the more intense labelling is mentioned by a dark line. Dark bars represent the average of A, B, C, D structures. (E) Actin (magenta) and E‐cadherin (cyan) staining detected 1.1 μm above the gelatin bottom using AiryScan module. Peaks represent labelling intensity of each molecule at this Z‐section.

In 12% of cells, both E‐cadherin and Actin organise into overlapping rings at the ventral cell surface prior to gelatin degradation (Figure 6A step 1 and Figure 6B). If actin rings are always associated with E‐cadherin rings, the reverse is not true. This strongly suggests that E‐cadherin ring structuration precedes actin assembly. Less frequently (3% of the cells) E‐cadherin/Actin rings are associated with starting degradation area (Figure 6A step 2 and Figure 6B), indicating that these structures enriched in both E‐cadherin and Actin represent invadopodia precursors. In 50% of the cells, a large area of degradation accumulates with punctiform actin labelling associated or not with an E‐cadherin staining. This suggests that after invadopodia maturation, E‐cadherin dispersion precedes actin disassembly (Figure 6A step 3 and Figure 6B).

We observed that a part of the E‐cadherin ring is localised less deeply than the actin ring in invadopodia (Figure 6C–E). Moreover, analysis of the localisation of the maximal labelling intensity of the 2 molecules confirms this observation (Figure 6C,D). Representation of the intensity of E‐cadherin and Actin staining using Airyscan acquisitions, for a section taken 1.1 μm from the bottom of the gelatin, shows that these rings are not blended into the background (Figure 6E). Taken together, these results demonstrate a structuring role for E‐cadherin during invadopodia formation.

## 4 Discussion

E‐cadherin was associated for years as a tumour suppressor. However, high levels of E‐cadherin expression have been demonstrated in various invasive and metastatic cancers with epithelial traits suggesting that E‐cadherin may have inefficient suppressive activity or, even worse, could promote metastasis instead of suppressing tumour progression [18, 19]. Moreover, studies demonstrated that E‐cadherin expression in E/M hybrid cells expressing E‐cadherin might confer collective migratory ability to tumour cells, allowing them to survive during transit and colonisation in distinct organs [19, 36].

To address the role of E‐cadherin in PDAC aggressiveness, we modulated E‐cadherin expression or activity in pancreatic cell models to analyse its effect on membrane protrusions so‐called invadopodia capable of local ECM degradation leading to cancer cell dissemination into surrounding tissues and blood vessels [20, 22, 31]. According to our data, the contribution of E‐cadherin to local ECM degradation can be summarised as follows: E‐cadherin is an early component of invadopodia. (1) E‐cadherin can be endocytosed and recycled back to the invadopodial membrane simultaneously with MT1‐MMP. Both Rab7 (transport to endocytic compartments) and/or Rab11 (dynamics of recycling endosomes) dependent pathways are required for this trafficking; (2) Once translocated into the immature invadopodia, E‐cadherin interacts with several structuring components, such as Arp2/3 and Actin; (3) In association with Actin, E‐cadherin forms a ring that precedes invadopodia degradative activity; (4) E‐cadherin–β‐catenin trans‐interactions at the invadopodial membrane suggest the establishment of new adherens‐like junctions, allowing actin tension required for the protrusion scaffold (see graphical abstract).

Invadopodia are hallmarks of various invasive cells [22, 37, 38, 39]. They have been extensively studied in cell culture and have now been detected in in situ tissue explants, tissue sections and in vivo models [23, 24, 40]. Invadopodia are supposed to represent promising therapeutic targets to prevent cancer metastasis [39]. Our ex vivo and in vivo results strengthen the physiological relevance of invadopodia in PDAC, as the two most used invadopodia markers (Cortactin and Tks5) colocalise preferentially at cell plasma membranes in close contact with the extracellular matrix. According to this, we postulate that BxPC3 cell lines are a suitable research model for invadopodia studies in PDAC.

We provide multiple lines of evidence that E‐cadherin is a key component of the invadopodial membrane. (1) E‐cadherin localises with both Cortactin and Tks5 close to the ECM surrounding tumour clusters in patient tissues; (2) a pool of E‐cadherin but not P‐cadherin is detected at the invadopodial membrane in the pancreatic BxPC‐3 cell line, pancreatic cancer primary culture and a cell line derived from an inflammatory breast cancer. Moreover, E‐cadherin interacts with the main components of invadopodia, including Cortactin, Arp3, Tks5 and MT1‐MMP. Furthermore, A pool of E‐cadherin is distributed in invadopodia when cell–cell contacts are reduced; (3) E‐cadherin is trafficked to the invadopodial membrane, as MT1‐MMP, through Rab7 and Rab11 recycling routes; (4) E‐cadherin is found in purified fraction of invadopodia; (5) Modulation of E‐cadherin expression is associated with invadopodia formation. Likewise, inactivation of E‐cadherin trans‐interaction in junctional complexes by drugs reversibly blocked invadopodia development; (6) E‐cadherin forms a ring that associates with the actin ring to form an invadopodia precursor structure. According to this, a recent study showed that E‐cadherin is a component of invasive protrusions and is necessary for the invasiveness of *Ras* ^*V12*^‐transformed intestinal epithelial cells in *Drosophila* [41]. Some other compounds of the junctional complexes, including tight junctions (ZO‐1) and gap junctions (Connexin 43), may regulate invadopodia formation [42, 43]. However, their ability to organise invadopodia has not been described.

The Arp2/3 complex polymerises actin filaments as branches from existing filaments and powers various cell processes including cell motility, endocytosis, vesicle trafficking and adherens junction stability [44, 45]. Its impact on actin polymerisation is critical for invadopodia‐based invasion by driving cell protrusions through the ECM and maintaining tight apposition of surface‐exposed MT1‐MMP with the ECM [46]. Here, we suggest a link between E‐cadherin and the Arp2/3 complex since E‐cadherin associates with Arp3 in invadopodia. Therefore, E‐cadherin and Arp3 depletion prevents the formation of the actin protrusion which normally sustains invadopodia.

From these results we postulate that E‐cadherin allows the establishment of membrane junctions in invadopodia structures. Cadherins have been described as participating in the formation of junctions within a single cell. For instance CDHR5 and CDHR2, indirectly anchored with the core actin bundle, are known to orchestrate microvillus crosslinking in intestinal enterocytes [47].

It is conceivable that other cadherins, such as N‐cadherin, CDHR5 or CDHR2 could be involved in invadopodia formation in cell models that do not express E‐cadherin, such as the mammary line MDA‐MB‐231. However, our study suggests that P‐cadherin is not involved in this process. further studies are needed to validate this hypothesis. E‐cadherin localization in invadopodia is not random; but is the result of active intracellular trafficking, including endocytosis and recycling via Rab7 or Rab11 vesicle‐dependent pathways. However, pathways involved in invadopodia activity, including Rab2A‐dependent vesicles and exocyst complex [48, 49] may also be involved in E‐cadherin trafficking to invadopodia. Although this is not the point of this article, further work is needed to define exactly how E‐cadherin is transported to invadopodia.

Three distinct but interconvertible migration programs (collective movement, mesenchymal and amoeboid migration) have been identified which differ in cell–cell and cell‐matrix adhesion, cytoskeletal organisation and mechanochemical tissue interactions [50]. It is easily conceivable that the migration mode of tumour cells could be regulated by the cadherin expressed, particularly in the case of cells co‐expressing several cadherins. According to this, we and other authors have shown that in tumour cells P‐cadherin is involved in collective migration [6, 7]. In this work, we showed that E‐cadherin regulates local ECM remodelling of more isolated cells. Further work is needed to determine how the nature of the cadherins can impact the plasticity of the migration program. To summarise, we demonstrated that E‐cadherin promotes pancreatic cancer cell invasion by regulating invadopodia formation. The pro‐invasive function of E‐cadherin and its related signalling mechanism need to be further explored. Importantly, these findings open new avenues towards uncovering innovative options for earlier diagnosis and anti‐invasive therapy of pancreatic cancer.

## Author Contributions

**Aurélie Dobric:** conceptualization (equal), investigation (equal), methodology (equal), validation (equal), visualization (equal), writing – original draft (equal). **Sébastien Germain:** investigation (equal), methodology (equal), validation (equal), visualization (equal). **Françoise Silvy:** investigation (equal), methodology (equal), validation (equal), visualization (equal). **Rénaté Bonier:** investigation (equal), methodology (equal), validation (equal), visualization (equal). **Stéphane Audebert:** conceptualization (equal). **Luc Camoin:** conceptualization (equal), investigation (equal). **Nelson Dusetti:** funding acquisition (equal), validation (equal). **Philippe Soubeyran:** investigation (equal), validation (equal). **Juan Iovanna:** funding acquisition (equal), investigation (equal), validation (equal). **Véronique Rigot:** conceptualization (equal), funding acquisition (equal), investigation (equal), methodology (equal), supervision (equal), validation (equal), visualization (equal), writing – original draft (equal). **Frédéric André:** conceptualization (equal), funding acquisition (equal), investigation (equal), methodology (equal), supervision (equal), validation (equal), visualization (equal), writing – original draft (equal).

## Ethics Statement

All experimental procedures involving animals were performed in accordance with French Guidelines and approved by the ethical committee of Marseille (agreement 50‐31102012). All Human pancreatic cancer samples used in this study were obtained as previously described (approval DC2013‐1857) [51]. Informed consent was obtained from all subjects.

## Conflicts of Interest

The authors declare no conflicts of interest.

## Supporting information


**Data S1.** Methods precisions for proteomic analyses.
**Figure S1.** Invadopodia characterisation in BxPC‐3 cells.
**Figure S2.** Videos of Invadopodia dynamic.
**Figure S3.** Controls for invadopodia imagery.

## Data Availability

The mass spectrometry proteomics data have been deposited to the ProteomeXchange Consortium (http://www.proteomexchange.org) via the PRIDE partner repository with the dataset identifier PRIDE: PXD017895. To access these data, reviewers can log in using the following information Username: reviewer_pxd021795@ebi.ac.uk and Password: Su16pLp6. Other data that support the findings of this study are available from the corresponding author upon request.
