# Supplementary material for: E‐Cadherin Is a Structuring Component of Invadopodia in Pancreatic Cancer
Source: J Cell Mol Med. 2025 May 14;29(9):e70608. doi: 10.1111/jcmm.70608 (PMC12077114; doi:10.1111/jcmm.70608)
Supplement: Supplementary file 1 — Data S1. Methods precisions for proteomic analyses. Figure S1. Invadopodia characterisation in BxPC‐3 cells. Figure S2. Videos of Invadopodia dynamic. Figure S3. Controls for invadopodia imagery. [file JCMM-29-e70608-s001.docx]

**Additional Data 1**

**Mass spectrometry analysis and data processing protocol.** Proteomes from E-cadherin depleted cells (BxPC-3 shEcad) were compared to control cells (BxPC-3 shCTRL) by label-free quantitative mass spectrometry analysis. 15 µg of each cell lysate was loaded on NuPAGE 4-12% Bis-Tris acrylamide gels (Life Technologies) to stack proteins in a single band that was stained with Imperial Blue (Thermo Fisher Scientific) and cut from the gel. Gel pieces were submitted to an in-gel trypsin digestion (Shevchenko *et al*, 1996). Peptides were extracted from the gel and dried under vacuum. Samples were reconstituted with 0.1% trifluoroacetic acid in 4% acetonitrile and analyzed by liquid chromatography (LC)-tandem mass spectrometry (MS/MS) using an Orbitrap Fusion Lumos Tribrid Mass Spectrometer (Thermo Electron, Bremen, Germany) with a nanoRSLC Ultimate 3000 chromatography system (Dionex, Sunnyvale, CA). Peptides were separated on a Thermo Scientific Acclaim PepMap RSLC C18 column (2 µm, 100A, 75 µm × 50 cm). For peptide ionization in the EASY-Spray nanosource in front of the Orbitrap Fusion Lumos Tribrid Mass Spectrometer, spray voltage was set at 2.2 kV and the capillary temperature at 275 °C. The Orbitrap Lumos was used in data-dependent mode to switch consistently between MS and MS/MS. The time between master scans was set to 3 seconds. MS spectra were acquired with the Orbitrap in the range of m/z 400–1600 at a FWHM resolution of 120,000 measured at 400 m/z. AGC target was set at 4.0e5 with a 50 ms maximum injection time. For internal mass calibration, the 445.120025 ions were used as lock mass. The more abundant precursor ions were selected, and collision-induced dissociation fragmentation was performed in the ion trap to have maximum sensitivity and yield a maximum amount of MS/MS data. Number of precursor ions was automatically defined along run in 3 s windows using the “Inject Ions for All Available parallelizable time option” with a maximum injection time of 300 ms. The signal threshold for an MS/MS event was set to 5,000 counts. Charge state screening was enabled to exclude precursors with 0 and 1 charge states. Dynamic exclusion was enabled with a repeat count of 1 and duration of 60 s.

Relative intensity-based label-free quantification (LFQ) was processed using the MaxLFQ algorithm from the freely available MaxQuant computational proteomics platform, version 1.6.3.4. Spectra were searched against the human database extracted from UniProt on the 1^st^ September 2020, which produced 20,375 entries (reviewed). The false discovery rate (FDR) at the peptide and protein levels were set to 1% and determined by searching a reverse database. For protein grouping, all proteins that could not be distinguished on the basis of their identified peptides were assembled into a single entry according to the MaxQuant rules. Statistical analysis was done with Perseus program (version 1.6.14.0) from the MaxQuant environment ([www.maxquant.org](http://www.maxquant.org)). Quantifiable proteins were defined as those detected in above 70% of samples in one condition or more. To obtain a normal distribution, protein LFQ normalized intensities transformed using base 2 logs. Missing values were replaced using data imputation by randomly selecting from a normal distribution centred on the lower edge of the intensity values that simulates signals of low abundant proteins using default parameters (a downshift of 1.8 standard deviation (s.d.) and a width of 0.3 of the original distribution). To determine whether a given detected protein was specifically differential, a two-sample *t*-test was done using permutation-based FDR-controlled at 0.01 and employing 250 permutations. The *p* value was adjusted using a scaling factor s0 with a value of 0.4. Analysis was done on biological triplicates, each run three times on mass spectrometers. The mass spectrometry proteomics data have been deposited to the ProteomeXchange Consortium via the PRIDE (Perez-Riverol *et al*, 2019) partner repository with the dataset identifier PXD021795.


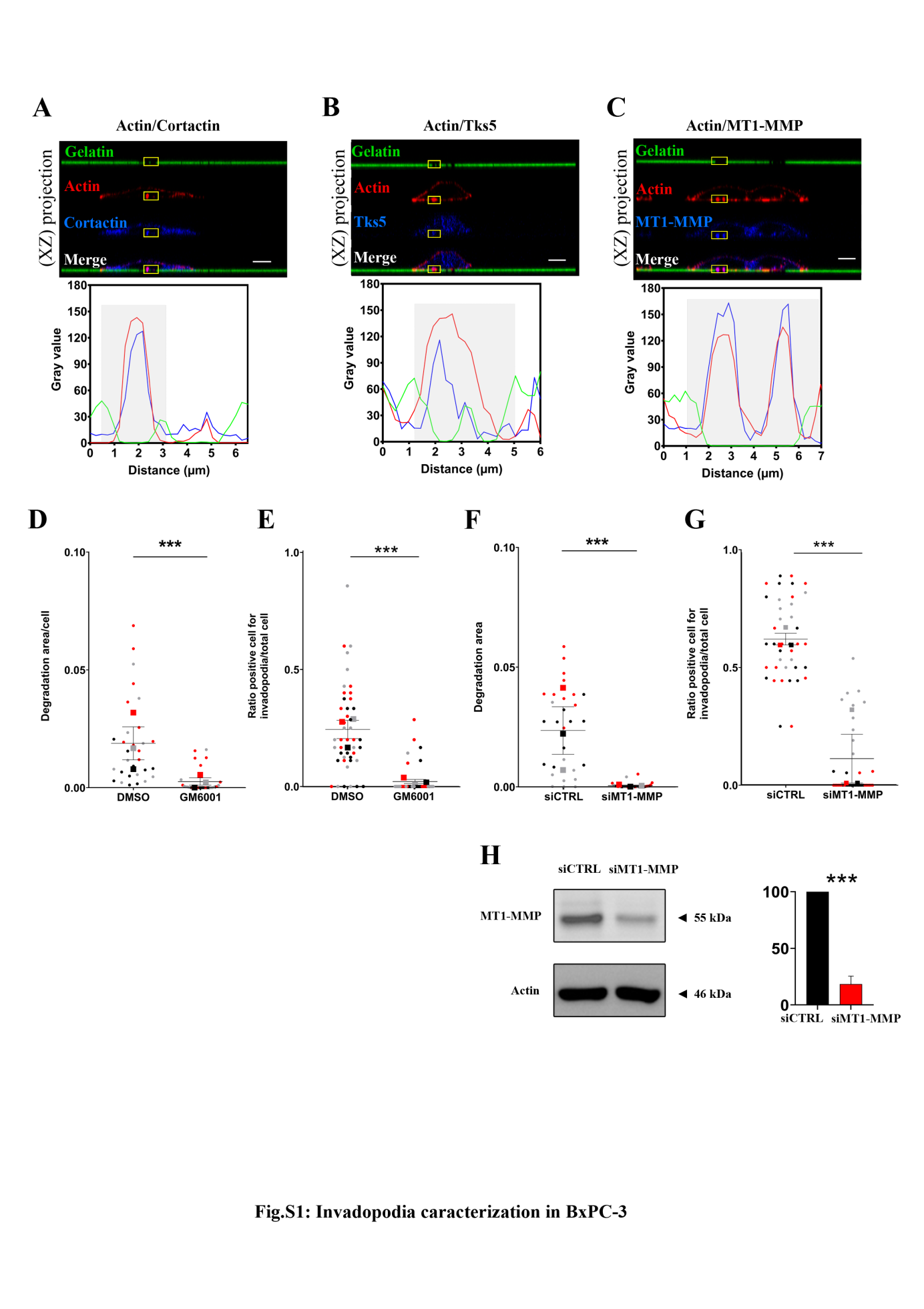


**Fig.S1: Invadopodia characterization in BxPC-3 cells.**

**(A-C)** BxPC-3 cells were plated for 16h onto FITC-labelled gelatin then fixed. Actin, Tks5 and MT1-MMP were immunostained. **(A)** Actin (red) and Cortactin (blue), **(B)** Actin (red) and Tks5 (blue), **(C)** Actin (red) and MT1-MMP (blue). Z-stack acquisitions were then performed. Top panels: Colocalization of actin spots with a degradation zone of the gelatin (black spot) represents active invadopodia. Scale bar = 2 µm. Bottom panels: Fluorescence intensity quantification of the region of interest indicated by the yellow square on the left panel. The gelatin degradation area is identified in grey. **(A-C)** A representative image of 5 experiments with 3 acquisitions for each (n=5).

Images in 2D view for (**A-C**) are available in ***Fig.S4A***

**(D)** Quantification of gelatin degradation area at the ventral surface of treated (GM6001 inhibitor) and control (DMSO-treated) BxPC-3 cells. **(E)** Ratio of positive cells for active invadopodia in treated (GM6001 inhibitor) and control (DMSO-treated) BxPC-3 cells. **(F)** Quantification of gelatin degradation area at the ventral surface of siCTRL or siMT1-MMP treated cells. **(G)** Ratio of cells exhibiting active invadopodia in siCTRL and siMT1-MMP treated cells... **(D-G)** 10 microscopic fields are quantified for each condition of the 3 experiments; Mean from 3 independent experiments are indicated with coloured squares Errors bars represent Mean ± SEM **(H)** Western blot analysis of MT1-MMP protein expression in BxPC-3 siCTRL and siMT1-MMP cells. BxPC-3 cells were treated for 48h with siRNA control (siCTRL) or siRNA against MT1-MMP (siMT1-MMP). MT1-MMP and actin were detected using specific antibodies. The graph represents the mean ± SEM from three independent cell transfections. n=3

**
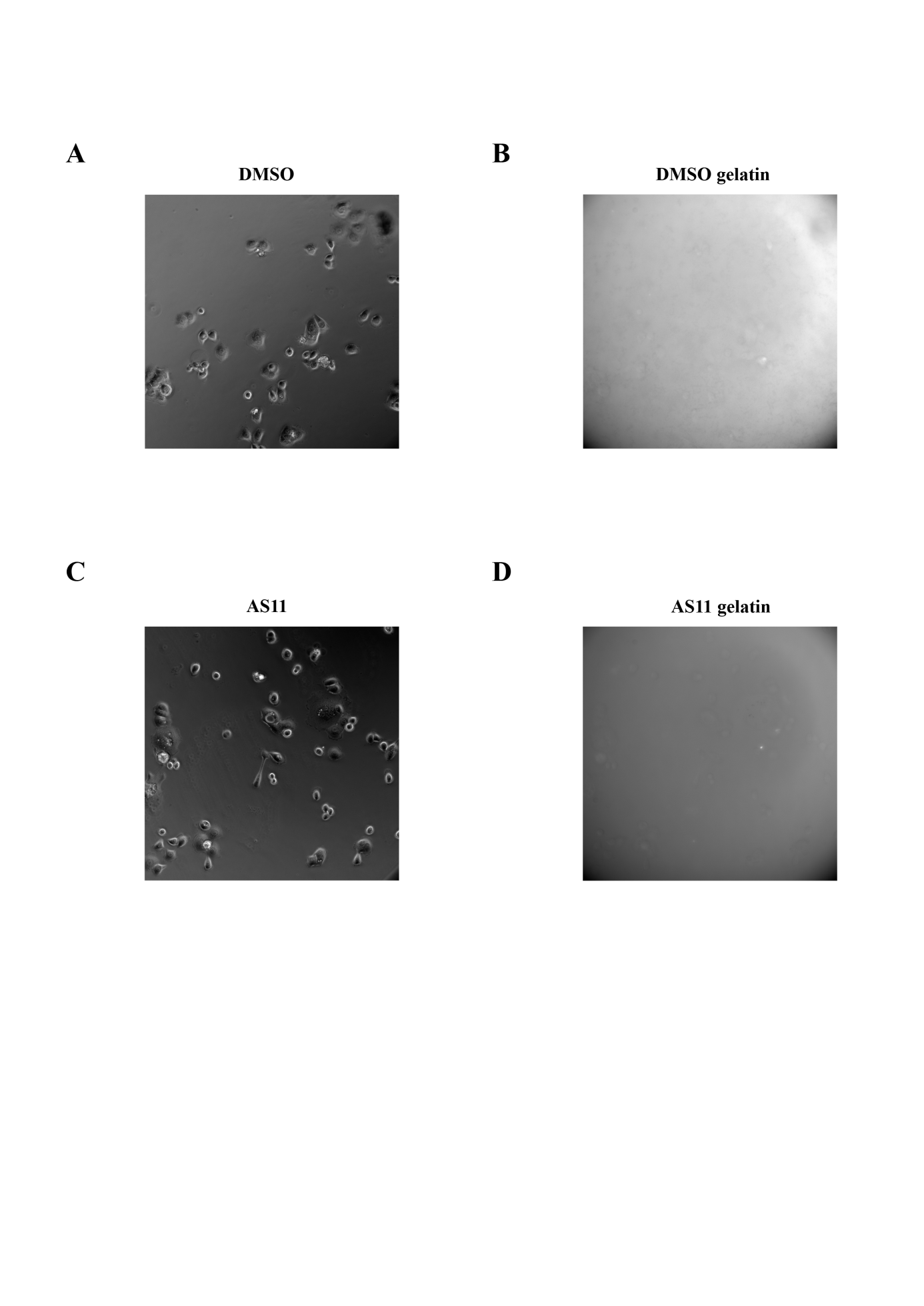
**

**Fig.S2: videos for invadopodia dynamic**

Invadopodia assays were performed using BxPC-3 shCTRL. Cells were seeded for 2h on coverslips coated with FITC-conjugated gelatin, then treated for 16h with DMSO (**A** and **B**) or AS11 (**C** and **D**). Cells were then washed and incubated in DMEM/10% fetal calf serum for an additional 24h period. Invadopodia formation was analysed by videomicroscopy by capturing images every hour, 8h after addition of the compounds. **A** and **C** represent bright field images; **B** and **D** represent gelatin degradation areas. The number of gelatin degradation zones appearing just below the cell body is estimated for each hour.

The graph (representative of an experiment carried out three times) is available in Fig. 4H

The 4 videos that constitute the Figure S1 are available via the following link:

https://amubox.univ-amu.fr/s/eBtpxfLDxDEbeZ2


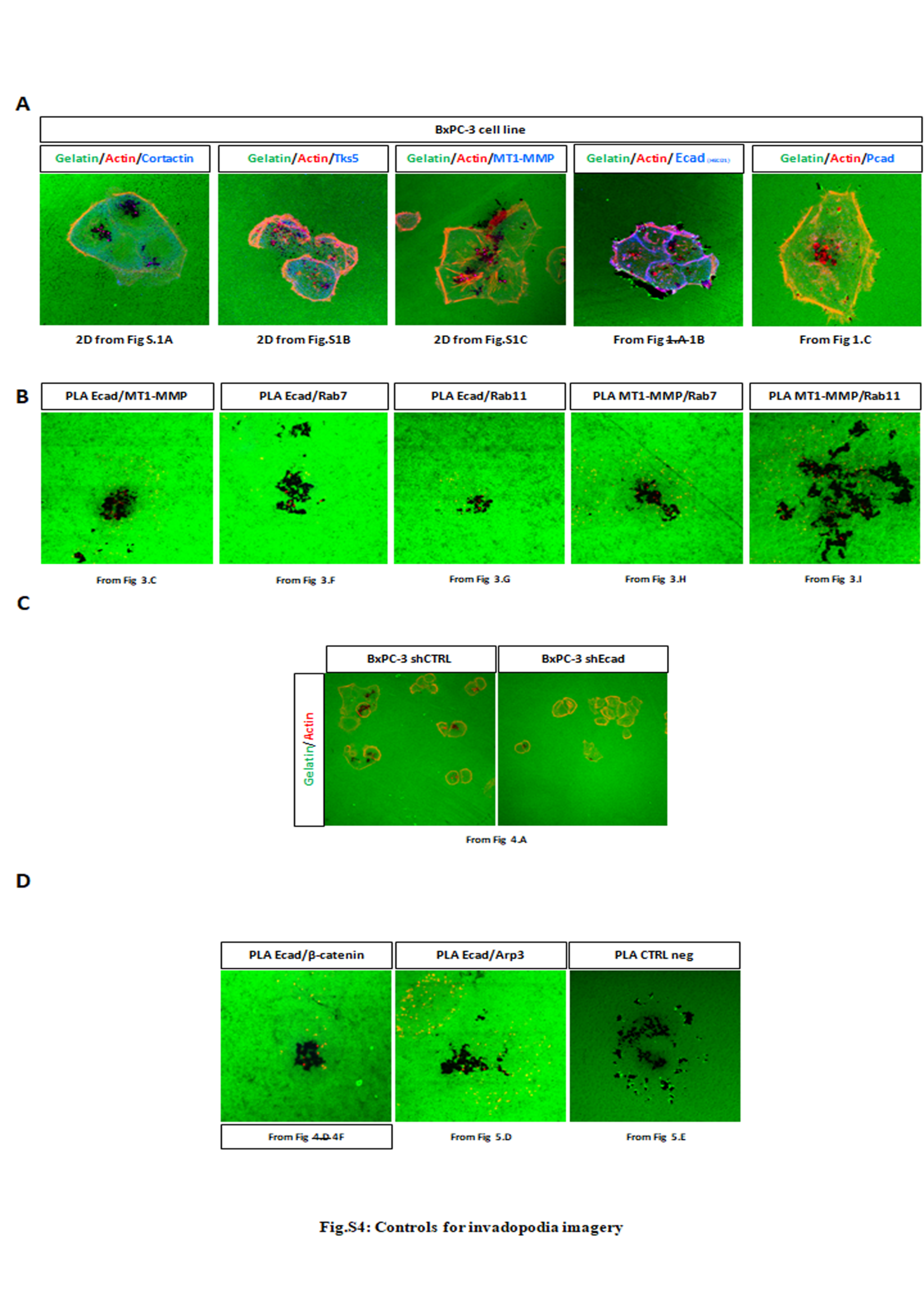


**Fig.S3: Controls for invadopodia imagery**

(**A**): 2D view of invadopodia labelling presented in Fig. 1 and Fig. S1.

(**B**): 2D view of invadopodia labelling presented in Fig. 3.

(**C**): 2D view of invadopodia labelling presented in Fig. 4A.

(**D**): 2D view of PLA labelling (presented in Fig. 4) and negative control of PLA (presented in Fig. 3 and 5).
